# Cross-Sectional Study of the Professional Quality of Life of Palliative Care Professionals during the COVID-19 Pandemic

**DOI:** 10.3390/healthcare12010004

**Published:** 2023-12-19

**Authors:** Adrià Campos i Arnal, Laura Galiana, Javier Sánchez-Ruiz, Noemí Sansó

**Affiliations:** 1 Department of Methodology for the Behavioral Sciences, University of Valencia, 46010 Valencia, Spain; acamiar@alumni.uv.es (A.C.i.A.); jasan4@alumni.uv.es (J.S.-R.); 2 Department of Nursing and Physiotherapy, University of the Balearic Islands, 07120 Palma, Spain; 3 Balearic Islands Health Research Institute (IDISBA), 07120 Palma, Spain

**Keywords:** compassion, professional quality of life, palliative care, healthcare professionals, compassion satisfaction, burnout, compassion fatigue

## Abstract

Background: The display of compassionate care by palliative care professionals is of the utmost importance to the patients, their families, and even to their own professional well-being. Lately and, especially due to the emergence of the COVID-19 pandemic, palliative care professionals have been subjected to greater pressures stemming from their work environment, organizational standpoint, and emotional sense of view. Not only have these factors made it harder for professionals to deliver compassionate care to their patients, but they have also decreased their overall well-being. The aim is to study how sociodemographics, workplace characteristics, internal resources, and the COVID-19 pandemic-derived pressures have affected the professionals’ capacity to perform compassionate care and their well-being while at the same time exploring the relationship between compassionate care and well-being. Methods: This study used a cross-sectional design with data gathered from Spanish palliative care professionals. The final sample was formed by 241 participants. They were surveyed about compassion, professional quality of life, well-being, sociodemographic data, working conditions, self-care, and coping with death competence, and the impact of the COVID-19 pandemic. The analyses used were descriptive statistics, bivariate tests, and the construction of a structural equation model. Results: Compassion was predicted by the ability to control their workload and the ability to cope with death. Burnout was predicted by age, workload, workload control, self-care, material resources, and changes in teamwork. Moreover, compassion, age, workload control, and changes in teamwork and self-care were shown to significantly predict compassion satisfaction. When it comes to compassion fatigue, different variables were shown to predict it, those being compassion, control over the workload, social self-care, and the ability to cope with death. Conclusions: Having a healthy lifestyle and an adequate social support system is key to maintaining professional well-being in the case of palliative care professionals. Inner resources such as the ability to perform self-care and the capacity to cope with death are of vital importance to taking care of these professionals. Thus, it would be beneficial to establish training programs focused on these aspects in the myriad of sanitary centers that perform these tasks, as these abilities are necessary to withstand the work-related pressures and, at the same time, be able to provide compassionate care for patients.

## 1. Introduction

### 1.1. Compassionate Care, Compassion, and Professional Quality of Life

Caring made from compassion, also known as compassionate care, is a fundamental dimension of the nursing profession and a vital component of high-quality care [1]. Compassionate care can be defined as how we relate to others when they are vulnerable. This includes taking responsibility for the other’s vulnerability and experiencing the emotional reactions that take place during the interactions with the other person [2]. According to the existing literature, compassionate care is a subjective experience that is based on a quality relationship, takes into account that the person providing care also connects with the other’s necessities, stems from the common human experience and the need to preserve the subject’s integrity while acknowledging their suffering and vulnerability and, finally, and also includes the ability to emotionally connect with the patient and different interpersonal capabilities [3,4,5,6]. Thus, care with/from compassion is a key component to the achievement of high-quality care.

Recently, compassion has been defined as the sensitivity to perceive suffering both in others and ourselves with the purpose of trying to prevent it or, at least, subdue it [7,8]. Moreover, it has also been defined as a response that aims to buffer people’s suffering and needs via the conceptualization of suffering as a common human experience and different relational tasks [9,10,11]. Compassion is also considered to be one of the six main elements that are vital to care provision [12]. Apart from this, other authors suggest that compassionate care requires the person to adopt an empathic attitude, which includes the ability to identify the other’s suffering and the capacity to attune with the other [10].

Different studies have underscored the essential role that compassion has in the professional quality of life of healthcare professionals [13,14] and that receiving compassionate care is one of the most important necessities for patients while, at the same time, rarely satisfied [15]. Compassion is associated with the professionals’ quality of life [16], the health of healthcare workers [17], and their well-being [18]. For example, self-compassion, which is the ability to be compassionate with oneself, has been found to play a protective role in burnout and compassion fatigue [14]. Furthermore, compassion and the well-being of healthcare workers have also been related [19,20].

Professional quality of life can be defined as the way a person feels about his/her job as a care provider [21]. This encompasses both the positive and negative aspects that may influence life’s quality and comprise different elements, such as burnout, compassion fatigue, and compassion satisfaction [21]. Professional quality of life is of paramount importance to palliative care professionals, among other factors like providing quality service and satisfying the patient’s needs [22,23,24,25]. The development of burnout is also associated with a worsening in the care provided by professionals, which, in turn, increases patients’ dissatisfaction [26] and leads to an increase in the number of medical errors [24]. As a result of burnout, in last place, three processes have been known to take place: emotional exhaustion, depersonalization, and a diminished perception of self-realization [26,27].

The definition of burnout provided by Maslach [28] conceptualizes it as a prolonged response to chronic emotional and interpersonal stressors at work that mainly comprise emotional exhaustion, cynicism, and/or depersonalization and a reduction in personal achievements. On the other hand, the term compassion fatigue is used to refer to the stress, exhaustion, or negative effect that stems from the relationship between professionals and patients [29,30]. Previous scientific literature points out that compassion fatigue carries along an increase in the difficulty to providing care and that it can be seen in changes throughout different areas of life, such as the social, physical, emotional, spiritual, and intellectual [29]. Nevertheless, different definitions of compassion fatigue produce alternative proposals for its dimensions. For example, Hotchkiss [31] went on to say that compassion fatigue is made up of two dimensions, those being secondary traumatic stress and exhaustion. As a consequence of compassion fatigue, professionals can feel drained of energy, indifferent toward their patients, apathetic, a decrease in work performance, an increase in thoughts about resigning, etc. [29,32]. As suggested by the model proposed by Stamm [21] for the professional quality of life, apart from its negative dimensions (i.e., burnout and compassion fatigue), there is at least one positive aspect: compassion satisfaction. Compassion satisfaction is the ability to receive gratification from providing care for others; thus, it can be conceived as the degree of joy that results from the experience of helping others while in healthcare [30,31]. Stamm [21] suggested that compassion satisfaction can be thought of as the positive part of caring for others.

These definitions show that professional quality of life is closely related to compassion. Moreover, evidence supports this fact. For example, professional quality of life has been proven to correlate positively with compassion satisfaction and to prevent the effects of compassion fatigue, exhaustion, or burnout [14]. Recent studies, such as the one by Mesquita-García et al. [13], have pointed out that for healthcare professionals, self-compassion relates both to an increase in the professional quality of life and a decrease in the perceived risk of exhaustion and secondary traumatic stress. Furthermore, self-compassion, as laid out by Galiana et al. [14], has a protective role against the development of exhaustion and compassion fatigue. Finally, self-compassion has also been related to other protective factors such as self-care and coping with death skills [14], variables previously related to professional quality of life [30,33,34].

### 1.2. Well-Being and Its Relation with Compassionate Care

Well-being has a myriad of diverse definitions [35], making it one of the most debated terms across scientific literature. Nevertheless, these definitions could be grouped into two main groups, those being hedonic and eudaimonic well-being. For example, Diener’s approximation [36] is more akin to the hedonic conceptualization, which is centered around subjective well-being. These types of definitions focus on the person’s positive affect and hedonic pleasure [37], as hedonic well-being is usually thought of as experiencing great levels of positive affect and pleasure while also experiencing low levels of negative affect. On the other hand, eudaimonic approximation suggests that well-being is not the result of external factors but rather the process of accomplishing and fulfilling one’s potential and life projects [38].

In the context of healthcare professionals, the study of well-being has gained importance over the last few years. As it has been previously noted, healthcare workers are specifically exposed to stress in their job and, especially when dealing with patients [29]. In the case of palliative care professionals, their job specifically involves dealing with stress-inducing situations that can affect the worker’s health and well-being on a daily basis [39]. The literature has shown that compassionate qualities/abilities are essential to the well-being of professionals in palliative healthcare [14,40]. Moreover, it has been pointed out that professional quality of life and compassionate care are closely related to the workers’ well-being as greater self-care in professionals translates into more compassionate care for patients and higher perceived levels of happiness for workers [14]. For example, two recent studies have proved this relationship between professional quality of life and well-being [14,40]. In the same direction, Lizano [41] showed that there is a negative impact on the professional’s well-being due to exhaustion. Other investigations have suggested that the professional quality of life can explain 60% of the variance in nurse’s well-being [40]. As shown by Sansó et al. [40], a decrease in the professional’s quality of life can have far-reaching consequences for their well-being. Based on these data, the professional’s quality of life seems to be a crucial variable when it comes to the well-being of healthcare professionals [40,42]. In this sense, greater levels of compassion satisfaction and lower levels of exhaustion can predict a higher well-being. This evidence is acknowledged in different studies in which burnout and compassion fatigue have been shown to have a negative impact on the well-being of palliative care workers [33,37,43].

### 1.3. Variables Related to Compassionate Care and Well-Being

In relation to compassionate care, some studies have presented evidence of its relationships with sociodemographic variables such as age, which, for example, has been related to a greater risk of exhaustion [44,45]. Indeed, age and working experience are associated with a greater risk of burnout [27]. This is because those with fewer years of experience tend to stress out while, at the same time, have fewer coping strategies, which leads to a greater risk of exhaustion [46,47,48]. However, some studies have not found these previously cited relationships between age, working experience, and exhaustion [49,50]. As it stands, there is no consensus in the present literature. When it comes to gender, many studies have put forward that being a woman is related to the professional quality of life [51], showing that women experience more exhaustion [25,52,53] and compassion fatigue [54,55]. Nevertheless, other studies have found greater levels of deterioration in men [45,52], and some others have not found a relationship between gender and professional quality of life at all [49,50]. Other variables that have been related to compassionate care are the type of profession and the type of unit one works in. For example, one study found that exhaustion was more prevalent in social workers [56]. Others have pointed out that palliative care professionals who work by commuting to the patient’s home are at a greater risk of developing psychopathological symptoms than those who provide care in a hospital setting [42,56]. On the other hand, research shows that working fewer hours, having clinical supervision, and being young and a woman are associated with a larger level of well-being [17,37,57]. Be that as it may, other studies suggest that there are no significant differences between genders (men/women) when talking about well-being in palliative care professionals [58].

The available resources, the existent organization, and the different demands associated with the job also have an impact on the professional quality of life of palliative care workers. According to some articles, psychological and emotional demands have an impact on the well-being of healthcare professionals [59] and the professional quality of life [36] and can lead to burnout [60,61]. In this same sense, other studies have demonstrated that performing palliative healthcare is a highly demanding activity [43,62]. Thus, it is possible that these dire working conditions could impact the professional quality of life and the self-care abilities of palliative care workers. Specifically, the scientific literature evinces that the specific work/task demands related to it, the presence of illness, and the suffering and death of patients and their families are variables highly related to the exhaustion levels in palliative care professionals [61,63,64]. Furthermore, it has also been shown that excessive job demands, whether from supervisors or organizations, are also related to exhaustion and/or burnout [47,65]. Additionally, plenty of studies have proved that greater working hours [66], too many administrative tasks [64], the working environment, administrative support, and the availability of support services and relationships with colleagues [47] are also related to burnout levels. In the case of palliative care professionals, it is common for them to work in small organizations, for many hours, or work on the weekends, which are factors related to greater exhaustion [48].

Other highly relevant variables that are related to compassionate care and the palliative care professionals’ well-being are the internal resources of the worker. Based on the quantity and quality of the self-care abilities of each professional, they will be able to provide compassionate care and cope with exhaustion, compassion fatigue, and compassion satisfaction accordingly. In this sense, these abilities, in turn, end up having an effect on the professional’s well-being. Resources such as resilience [67], self-care [68], and physical activity [69] can play a vital role in dealing with job demands, with them being related to lower exhaustion. According to Hotchkiss [31], healthcare professionals who use frequent and multiple self-care strategies have a greater professional quality of life. Every type of self-care (physical, psychological, or social) can be considered a strong protective factor against burnout [30], with the use of supportive relationships [70] and conscient relaxation [31,69] being of great importance. In this sense, mindfulness has been related to lower burnout [71]. In fact, mindfulness has also been related to compassion as, via the training of compassion, there is an increase in self-consciousness, the ability to regulate one’s own emotions, and resilience [72]. Moreover, different studies propose that carrying out self-care techniques promotes an increase in well-being [31,33,73]. Based on this, the practice of meditation and different self-care activities that increase compassion, self-compassion, and emotional regulation are related to lower burnout [42]. Finally, the professional’s ability to cope with death is also a key variable in the informed professional quality of life. One example of this is the study carried out by Sansó et al. [30], in which it was evinced that this capacity is negatively related to burnout and compassion fatigue while having a positive relationship with compassion satisfaction. These results have been replicated in other more recent studies, such as Galiana et al. [14].

### 1.4. COVID-19 Pandemic and Its Impact on Palliative Care Professionals

The COVID-19 pandemic has had a remarkable impact on healthcare workers [70,74] at many levels: emotionally, in their job routine and demands, their workload, etc. [70,75,76]. In a study carried out by Nestor et al. [77], it was shown that the heaviest load and demands were placed on those professionals whose jobs consisted of providing prolonged, direct, and intimate care to the patient. Other identified factors of COVID-19 that heavily affected the professionals’ quality of life have been the shortages of equipment and personnel, the great risk of infection, the minimum reinforcement, the lack of equipment, isolation, and a lack of support systems [78]. Moreover, the professionals exposed to these factors had their workloads enlarged, were put in vulnerable positions, and suffered from burnout, distress [70], anxiety, lack of sleep, and depression [53].

In general, healthcare workers have been suffering from increased pressure since the start of the COVID-19 pandemic. That is partly due to the fact that these workers had to remain in direct contact with different coronavirus cases, which made them more susceptible to infection and developing mental health problems [53,79]. In fact, the majority of them became infected during the pandemic, and some of them even died [80,81]. Being preoccupied with their coworkers’ and their own health and seeing in their own eyes the death of some of them can increase distress and exhaustion/burnout [82]. Taking this into account, COVID-19 brought about an increase in healthcare workers’ anxiety, distress, burnout [70,77,83], and depression rates [53,84]. In this sense, Lluch et al. [51] pointed out that professionals’ vulnerability to burnout and compassion fatigue has increased due to the cropping up of COVID-19. Furthermore, COVID-19 has also affected palliative care workers’ mental health [85].

### 1.5. Aim of the Study

Even though there is plenty of evidence that supports the relationship between compassionate care, defining this as the ability to maintain adequate levels of compassion and an adequate professional quality of life, and the healthcare professionals’ well-being, there is much less literature that focuses on the specific field of palliative care workers. This fact is all the more important when the impact of the COVID-19 pandemic is taken into account, whose effects, although greatly documented in general healthcare workers [63], are much scarcer when it comes to palliative care professionals. In this same sense, few studies have carried out investigations on this topic while using multivariate approaches, something that limits the evidence available and fails to provide a holistic point of view of the processes involved in compassionate care and well-being.

In this context, the present study’s objective is to gain insight into how the sociodemographic characteristics, the working conditions, internal resources, and the derived crisis from the emergence of COVID-19 have affected the palliative care workers’ ability to provide compassionate care and well-being while at the same time studying the relationships between compassionate care and well-being. To achieve this, different variables were studied in a sample of Spanish palliative care workers one year after the start of the COVID-19 pandemic.

## 2. Materials and Methods

### 2.1. Design

The current study is of a cross-sectional nature, in which the sample was made of Spanish palliative care workers. The data was gathered using an online survey sent by email to those registered in the Spanish Palliative Care Society [SPCS] between the months of March and April of 2021. Answers were then downloaded from SurveyMonkey (SurveyMonkey.com). Participation was strictly voluntary, and to take part in the study, the subjects had to sign an informed consent waiver.

### 2.2. Participants

The survey was sent to a total of 338 palliative care professionals who were registered in the directory of the Spanish Palliative Care Society [86]. Professionals were contacted by email via two messages in the span of three weeks. Every invitation to participate included instructions to share the survey with other colleagues. The minimum required sample size was not calculated. Instead, the general rule of a minimum of 200 subjects was used, as it is common for Structural Equations Modeling [87]. To be included in the study participants had to be healthcare workers who, at the time of the survey, were working with patients at the end of their life.

A total of 278 professionals answered the survey, so that the response rate came out to be 82.24%. Nevertheless, as the participants were instructed to share the survey with other colleagues, this index is an approximation. After discarding those professionals who did not meet the inclusion criteria and/or had missing data on the main variables (compassion, professional quality of life, and well-being), the resulting number of subjects was 241.

### 2.3. Variables and Measurement Instruments

Compassion was assessed via the compassion subscale of the Dispositional Positive Emotion Scale (DPES) [88]. This subscale is composed of five items that use a Likert-type scale of five positions. As the scale was not validated in Spanish, we proceeded to translate and validate it. For the translation of the scale, we used the backward and forward translation process. First, the scale was translated into Spanish by a professional native; it was then translated back into English by another native professional. The final version was revised by four experts in psychometrics, cross-cultural instrument development and validation, and clinical psychology. All of them judged the instrument to adequately measure compassion, and no revisions from the original backward-forward translation were made. Evidence of validity was gathered by testing a confirmatory factor analysis, in which a latent factor of compassion was hypothesized. Results showed evidence of excellent fit: *χ^2^*(5) = 19.91 (*p* < 0.01), CFI = 0.97, TLI = 0.95, SRMR = 0.05 and RMSEA = 0.11 [0.64,0.17]. The reliability of the scale was 0.83.

The professional quality of life was evaluated via the short version of the Professional Quality of Life Scale (Short-ProQOL) translated into Spanish by Galiana et al. [89]. This scale is made up of three dimensions (compassion satisfaction; compassion fatigue; and burnout), which have three Likert-type items, each graded from (1) never to (5) very commonly. The dimension of compassion satisfaction can be considered “low” for scores lower than 10, “mid or average” for scores between 11 and 13, and “high” for scores of 14 or higher. When it comes to burnout scores, scores of 6 and lower are considered to be “low”, between 7 and 8 can be considered “average”, and scores of 9 or higher can be thought of as “high”. Finally, for compassion fatigue, scores of 4 or lower are considered “low”, 5 is considered to be “intermediate”, and from 6 onward, “high” [90]. Reliability estimates were 0.83 for the compassion satisfaction scale, 0.79 for compassion fatigue, and 0.85 for burnout.

To assess well-being, the Personal Well-being Index (PWI) in its Spanish translation made by Pérez-Belmonte et al. [58] was used. This scale measures personal well-being via eight items, which use a Likert-type scale with 5 points that range from (1) extremely unsatisfied to (5) very satisfied. This scale showed adequate psychometric properties with a reliability index of 0.91.

Moreover, questions concerning sociodemographic variables such as gender (man/woman) and age (in years) were added. For the measurement of working resources, they were assessed via two indicators: “I have an excessive workload” to evaluate the overall quantity of work, and “I have control over my own workload”, which was used to inquire about the professionals’ ability to manage their workload (workload control). Both of these questions had a response format of 3 points that ranged from 0 (never) and 3 (always).

Inner resources included in this study, those being self-care and the professionals’ ability to cope with death, were also measured. Self-care was assessed via the Professional Self-Care Scale (PSCS) in its Spanish translation [91]. This scale evaluates three self-care domains, those being physical, psychological, and social self-care. Each one of these subscales is composed of three items that use a Likert-type format that ranges from 1 (strongly disagree) to 5 (strongly agree). Moreover, all of them showed psychometric adequacy, with reliability indexes being 0.83 for physical self-care, 0.91 for psychological self-care, and 0.75 for social self-care. To assess the professionals’ ability to cope with death, the short version of the Coping with Death Scale (CDS-S) in its Spanish translation made by Galiana et al. [92] was used. This instrument evaluates the professionals’ competencies in dealing with death and their knowledge about the preparations required via nine items that are scored on a Likert scale from 1 (strongly disagree) to 5 (strongly agree). The reliability estimate for the present study was 0.91.

Finally, the COVID-19 pandemic’s impact was measured via the following indicators: (1) equipment during the health crisis: “Since the beginning of the pandemic, have you had the necessary material resources (masks, gloves, IPEs)?” with a yes/no format; (2) regarding changes in the professionals’ workload derived from the pandemic: “Since the beginning of the pandemic, has your workload changed?” with a Likert-type format that ranged from 1 (has considerably decreased) to 5 (it has considerably increased); (3) regarding changes in teamwork: “Since the beginning of the pandemic, has teamwork changed?” (for example, the coordination between the different members of the team or the participation in the decision-making processes), with a Likert-type format that ranged from 1 (has considerably worsened) to 5 (it has become considerably better); and (4) regarding if surveyed professionals had to provide care for COVID-19 patients: “Have you had to provide care for COVID-19 patients?” with a yes/no answer format.

### 2.4. Statistical Analysis

**Figure 1:**
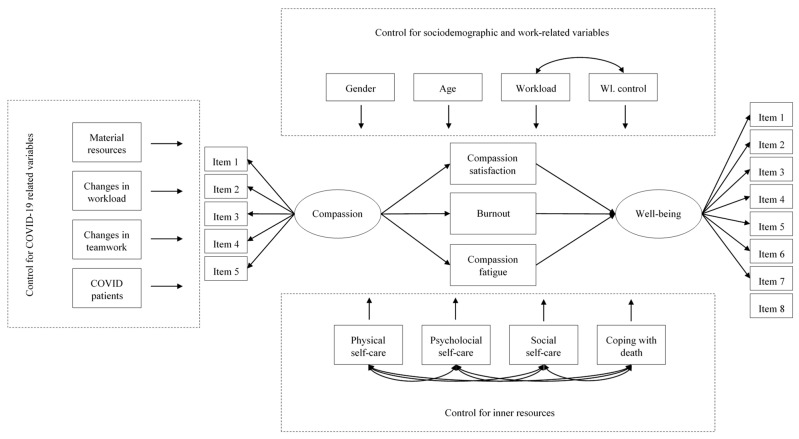
Hypothesized and tested model.

The first analyses used in this study were descriptive statistics for the main variables. They are quantitative data, averages, standard deviations, minimum and maximum scores, skewness, and kurtosis. Secondly, to study the relationship between compassion, professional quality of life, well-being, and the selected sociodemographic variables, *t*-tests were used for gender and correlation coefficients for the relationships with age. In the case of the work environment-related variables, as they can be considered semi-quantitative, Spearman’s correlation coefficients were used. For the internal resources, Pearson’s correlation coefficients were used. Finally, to study the impact of the COVID-19 variables, *t*-tests were used for the qualitative items (those being material resources and providing care for patients with COVID-19) and Spearman’s correlations for the semi-quantitative (i.e., changes in workload and teamwork). Finally, a complete structural equations model was hypothesized and specified. This can be seen in Figure 1.

As can be seen in Figure 1, the hypothesized model proposes that multiple variables can predict the main constructs of the study, those being compassion, professional quality of life, and well-being. Compassion and well-being will be modeled as latent factors, whereas the three dimensions of the professional quality of life and the rest of the predictive variables will be included as observed variables. Moreover, all the effects that the sociodemographic variables, internal resources, the impact of COVID-19, and the working conditions can exert on all of the dimensions that make up compassionate care and the professionals’ well-being will be both hypothesized and estimated. Additionally, it was hypothesized that the professionals’ ability to perform compassionate care will influence their professional quality of life, which in turn will lead to the latter affecting their overall well-being. Furthermore, the correlations between the working conditions and internal resources will also be estimated. To evaluate the goodness of fit, different indexes will be used: Chi-square, Comparative Fit Index (CFI), and the Root Mean Square Error of Approximation (RMSEA). The next cut-off points were selected as indicatives of a good fit for the model: CFI greater than 0.90 (better when greater than 0.95) and RMSEA smaller than 0.08 (better when smaller than 0.06) [93]. The model was estimated using robust maximum likelihood, which uses robust corrections for standard errors and goodness of fit indexes (weighted least square mean and variance-corrected, WLSMV), the recommended procedure for ordinal and non-normal data [94,95]. To perform the aforementioned statistical analyses, SPSS version 28 [96] and Mplus version 8.4 [97] were used.

### 2.5. Ethical Considerations

The participants of this study did so voluntarily and anonymously. The investigation complies with the ethical principles established in the Helsinki Declaration of the World Medical Association. All participants signed an informed consent present in the survey to authorize researchers to use and treat their data. Nevertheless, they could also retire this consent at any given moment and without consequences. This study was also approved by the Ethical Research Committee of the University of the Balearic Islands (115CER19).

## 3. Results

### 3.1. Participants’ Characteristics

The average age was 45.34 (SD = 10.92), with 77.2% (*n* = 186) of the surveyed being women. In terms of the work-related variables, the participants’ median of their “control over their own workload” was shown to be 2.00. This is proof of the excessive workload that this group experiences but, at the same time, of their ability to manage their own workload. Averages for the self-care and ability to cope with death dimensions were shown to be intermediate to high, with the lower scores being for psychological self-care (M = 2.97; SD = 1.05) and the highest for competency in coping with death (M = 3.96; SD = 0.63). Finally, when it comes to the COVID-19 experiences, the majority of participants answered that they had access to the resources and materials necessary to work (*n* = 163; 67.6%) and that they had to provide care for COVID-19 patients (*n* = 216; 89.6%). Moreover, the grand majority of them pointed out that their workload had increased significantly since the start of the pandemic (Md = 5.00; IR = 0.50) and that their working conditions, such as teamwork, had not changed (Md = 3.00; IR = 1.00). For more details, see Table 1.

### 3.2. Main Variables’ Characteristics

Firstly, descriptive statistics of the main variables were calculated. When it comes to compassion, the average score was 4.41, which implies large levels of compassion for others due to the DPES scale being graded from 1 to 5. For the professional quality of life, following the directions given by Galiana et al. [90] average values were found for the subdimensions of compassion satisfaction and burnout and large levels for the compassion fatigue subscale. Finally, the average for well-being was 3.95, a scale which was also graded from 1 to 5. All the values for univariate skewness and kurtosis for all the variables analyzed were satisfactorily within conventional criteria for normality (−3 to 3 for skewness and −10 to 10 for kurtosis), according to the guidelines suggested by Kline [87]. For more information, Table 2 can be consulted.

### 3.3. Relationships between Compassionate Care, Well-Being, and Sociodemographic Characteristics

To study the relationship between compassion, professional quality of life, and well-being with gender, *t*-tests will be used. These did not show differences between men and women in the aforementioned main variables (Table 3 can be consulted for the results).

When it comes to the relationship that age can have with the main variables, only one relationship proved to be statistically significant. In this case, the relationship between age and burnout showed that the younger professionals experienced greater burnout levels (results in Table 4).

### 3.4. Relationships between Compassionate Care, Well-Being, and Working Conditions

To study the relationship between compassionate care, well-being and workload and workload control, Spearman correlation coefficients were used. As can be seen in Table 4, the workload significantly and positively correlated with burnout and compassion fatigue. The control of their workload, on the other hand, correlated positively and significantly with compassion satisfaction and general compassion levels, with the greatest scores of these being exhibited by those professionals with the highest control over their own workload. Moreover, workload control also showed a negative and statistically significant correlation with compassion fatigue. With this in mind, it can be concluded that the greater the professionals’ control over their own workload, the lower the levels of compassion fatigue and burnout.

### 3.5. Relationships between Compassionate Care, Well-Being, and Internal Resources

The relationship between compassionate care, well-being, and the professionals’ internal resources was assessed via Pearson’s correlations. These can be seen in Table 4. The results pointed out that compassion shared positive and statistically significant correlations with social self-care and coping with death. In the case of compassion satisfaction, results suggested that it was positively and significantly correlated with all the self-care subdimensions and the ability to cope with death. This same pattern can be seen in well-being. On the other hand, burnout and compassion fatigue showed the opposite results, being negatively and statistically significantly correlated to all of the measured internal resources.

### 3.6. Study of Compassionate Care and Well-Being in Relation to the Clinical Experience during the COVID-19 Pandemic

To study the relationship between compassionate care, well-being, and professional quality of life with the clinical experience during the COVID-19 pandemic, *t*-tests and Spearman correlations were used. Firstly, *t*-tests were calculated to compare the averages of the main variables while dividing the participants between those who had the necessary resources to work correctly during the COVID-19 pandemic and those who did not. As can be seen in Table 5, the only difference between groups was found in burnout levels, where those who had the necessary resources experienced lower levels of burnout in comparison to those professionals who did not.

In terms of the effect that changes in workload might have exerted, this variable showed a positive and statistically significant Spearman correlation with burnout levels. In this case, in those professionals who experienced a great increase in their workload, their burnout levels increased accordingly. This can be seen in Table 4.

On the other hand, the changes in teamwork related to the COVID-19 pandemic measured by Spearman correlations showed that positive punctuations (which suggest that positive changes happened) were related to greater compassion satisfaction, whereas lower scores (negative changes or changes in teamwork for the worse) were related to greater burnout scores. For more detail, Table 4 can be consulted.

Finally, multiple *t*-tests were used to explore if there were significant differences in compassion, professional quality of life, and well-being between those professionals who had to provide care for COVID-19 patients and those who did not. As can be seen in Table 6, there were no statistically significant differences in none of the main variables.

### 3.7. Results of the Structural Equation Model

The hypothesized, specified, and tested Structural Equations Model showed adequate goodness of fit: *χ^2^*(266) = 443.15 (*p* < 0.01), CFI = 0.94, TLI = 0.91, SRMR = 0.15, and RMSEA = 0.05 [0.04,0.06].

In terms of the measurement part of the model, Table 7 shows the factor loadings for each of the modeled factors. The factor loadings were adequate for two both of the latent variables, varying from 0.44 (item 1) to 0.85 (item 3) for compassion; and between 0.61 (item 8) and 0.87 (item 1) for well-being. Details can be found in Table 7.

When it comes to the control variables, which include sociodemographic data, variables related to the working conditions, variables concerning the impact of the COVID-19 pandemic, and the professionals’ internal resources, their relationships with compassionate care and well-being can be seen in Table 8. As can be seen in Table 8, workload control and the ability to cope with death positively predicted compassion. This means that those professionals with more control over their workload and greater ability to cope with death were also those who exhibited greater compassion levels.

Compassion satisfaction was predicted by age, control over one’s own workload, and the positive changes in teamwork. As it stands, those older palliative care professionals who had a greater perceived control over their workload and who experienced positive changes in teamwork were also those who exhibited the larger amounts of compassion satisfaction.

Burnout was shown to be predicted by age, with greater levels of burnout being experienced by those younger; workload, with those professionals who had greater workload suffering from more burnout; control over one’s own workload, with those who had greater perceived control exhibiting less burnout; material resources, with greater burnout in those professionals who suffered from a lack of resources; changes in teamwork, where those who perceived the existence of changes for the worse experienced greater burnout; and social and physical self-care with higher burnout scores being related to lower levels of self-care (social and physical).

Compassion fatigue was predicted by the control over the own workload, social self-care, and the ability to cope with death, with greater levels of compassion fatigue being found in those professionals with lower levels on these cited variables.

Finally, well-being was positively and significantly predicted by workload, psychological, and social self-care; this means that those professionals with greater levels of workload, psychological, and social self-care were shown to have the greatest well-being from the sample.

**Figure 2:**
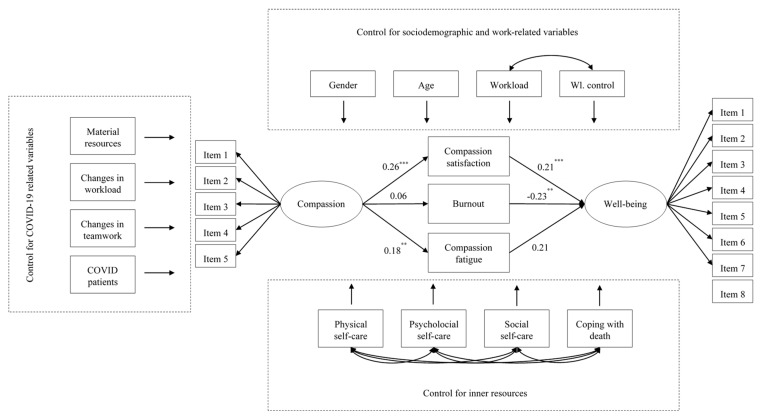
Relations among compassion, professional quality of life, and well-being in the structural equation model. ** *p* < 0.01; *** *p* < 0.001.

In terms of the relationship between compassionate care and well-being, the model suggested that compassion positively predicted compassion satisfaction and compassion fatigue. Compassion satisfaction and exhaustion levels were shown to be statistically significant predictors of well-being, with the first being positively related and the latter negatively related to it. The estimations are showed in Figure 2. In sum, the model explained 13% of the compassion variance (*R*^2^ = 0.13; *p* < 0.01), 21% of compassion satisfaction (*R*^2^ = 0.21; *p* < 0.01), 46.1% of burnout (*R*^2^ = 0.46; *p* < 0.01), a third of the variance of compassion fatigue (*R*^2^ = 0.34; *p* < 0.01), and more than half of well-being’s variance (*R*^2^ = 0.52; *p* < 0.01).

## 4. Discussion

The objective of the present study was to explore how the sociodemographic variables, the working conditions/characteristics, the internal resources, and the changes generated during the COVID-19 pandemic affected the relationship between the ability to perform compassionate care and well-being. To answer this, a series of relations were proposed, being based on the existent literature, which will guide the following discussion of the results obtained.

With regard to the role of age, it was found both in the bivariate associations and in the structural equation model that the more the age increases, the more the levels of burnout decrease. These results resemble others previously found in the literature [25,45,52,98], which found that there is a relationship between age and burnout risk. This would mean that the age per se diminishes burnout, also when variables such as job conditions are controlled for. The same happened with compassion satisfaction, with greater levels found with age. These results could be related to greater professional experience, as the older the professional is, the more years he or she has worked. However, it could also be hypothesized that informal education, such as courses that professionals undertake during their professional careers, can be a preventive factor for the quality of life. Future studies exploring these hypotheses would be welcomed. When it comes to the rest of the variables, no relationship with age was found.

Regarding gender differences, no differences based on gender were found at the bivariate level nor in the structural equation model. As it stands, previous research is not concluding in this regard, as others previously found gender differences in compassionate care [51] or well-being [17,37,71], whereas others have not [54,58]. However, these results should be taken into account with caution, as the total number of men present in the study was very small and could be a sign of a deficient sample size for this subgroup. In any case, in the studied sample, the professionals’ gender does not seem to play a crucial role in the compassionate care processes and well-being of the palliative care workers.

When it comes to the working conditions, based on the resulting data, statistically significant relations were found between workload, burnout, and well-being. In this sense, when the palliative care professionals’ workload increases, the risk of burnout and the informed well-being do as well. Whereas the relation with burnout was expected, the positive relation with well-being is counterintuitive. This can be partially due to the perception of a greater workload as a vital objective or a source of purpose for palliative care workers. When it comes to the professionals’ control over their workload, it was shown that the greater the professionals’ control over their workload, the higher the scores on compassion and compassion satisfaction while, at the same time, the lower the levels of burnout and compassion fatigue. These results were expected as the previous literature has shown the impact that control over the own workload can have on the professional quality of life [42,64,66]. However, it is the first time such a result is pointed to compassion. This result highlights the need for professionals to control their workload in order to maintain their compassionate capacity.

Regarding the role of inner resources, the structural equation model only showed one statistically significant and positive relationship between coping with death and compassion. In comparison to previous studies [72], the results obtained in the presented model suggest that after controlling for the professionals’ ability to cope with death, the self-care strategies are not relevant as predictors of compassion. This is in line with the recent results obtained by Galiana et al. [14] about the relationship between coping with death and compassion. Given this, it can be concluded that the key to owning the patients’ suffering is being able to deal or cope with death, an ability that grants palliative care professionals the capacity to cope with this suffering and provide better care for the patient. As regards the prediction of professional quality of life, self-care was key for predicting burnout in its dimensions of physical and social self-care, and social self-care and coping with death predicted compassion fatigue. These results go in line with previous literature, which has pointed out that the absence of self-care has been related to a greater compassion fatigue risk [99,100] and burnout [99], with healthcare professionals with multiple self-care strategies having a greater quality of life [31]. Given this, concerning the negative branch of the professional quality of life (burnout and compassion fatigue), the ability to cope with death and, especially, social and physical self-care are of vital importance. When it comes to the prediction of well-being, only psychological self-care and social self-care were significant predictors. These results concerning the link between well-being and psychological self-care line up with previously found evidence [31,33,73,101]. Although no specific relationships have been found between coping with death and well-being, it has been shown that the ability to cope with death improves the professional quality of life [14] and that, in turn, the latter is related to the professionals’ well-being [40,42], as it will be discussed a few lines ahead. These results could be pointing to the fact that there may be an indirect effect of coping with death on well-being that is mediated by the professional quality of life: coping with death produces an increase in general compassion, and this leads to greater compassion satisfaction, lower compassion fatigue, and, at the end of the chain, these abilities have an impact on well-being.

As for the impact of the COVID-19 pandemic on palliative care professionals’ compassionate care and well-being, this did not affect professionals’ compassion. Professional quality of life, in turn, was affected by changes due to the pandemic: positive changes in teamwork increased compassion satisfaction, and the absence of material resources and negative changes in teamwork increased burnout. These results are congruent with the existent previous literature [70]. Based on this, when teamwork worsens, a decrease in the quality of life takes place via lower compassion satisfaction and greater burnout indexes. These results are consistent with previous literature, as it has been found that teamwork has great importance on the professional quality of life when it comes to healthcare workers [47]. In the case of compassion and well-being, it is worth mentioning that they were not affected by changes in teamwork. With that in mind, the effect that changes in teamwork plays seems to be restricted to the professional context, not being able to affect overall compassion and well-being but only the professional quality of life. However, some of the results were unexpected, such as the absence of the effect of changes in workload over burnout. These results can be interpreted in various ways. For example, it could be that compassionate care may not heavily depend on the workload and its changes but rather on the control that professionals have over their schedule. In fact, as discussed previously, it appears that the professionals’ capacity to distribute their workload is more important than the overall volume of work. Another hypothesis could be that, taking into account the results obtained in the structural equations model, other variables may play a predictive role in the professional quality of life, such as self-care and/or coping with death. In any case, these are mere speculations, as these results should be further looked into. Regarding providing care for COVID-19 patients, the absence of effect on any variables goes against previously found evidence [53,77,79], which suggests that having to provide care for COVID-19 patients was related to a lower quality of life and mental health problems. In this sense, it can be hypothesized that it is not necessarily dealing with COVID-19 patients that caused these results but rather the conditions that cropped up as a result of it (lack of material resources, changes in teamwork) that would lead to a deterioration in the professional quality of life.

When it comes to the relationship between compassion and professional quality of life, the model showed significant and positive relationships between compassion and satisfaction, as well as between compassion and compassion fatigue. The obtained results are coherent with some of the existing literature, which has found that compassion is related to professional quality of life [14,16]. These results could point to a clear process of how to work with compassionate care: improving the professionals’ ability to cope with death will result in an increase in their compassion capabilities, which in turn would allow them to feel more compassion satisfaction and, at the same time, would also produce greater fatigue compassion. Nevertheless, other studies have suggested that the relationship between compassion with burnout and compassion fatigue may be negative. For example, Mesquita García et al. [13] showed that, in that study, only a positive relationship between compassion and compassion fatigue was found, which was the opposite sign of what was expected. This result would indicate that the compassionate capability of professionals is, at the same time, a necessary tool for the display of compassionate care and a risk factor for suffering from compassion fatigue: the more compassionate the professional is, the more he/she can suffer from compassion fatigue. In this sense, it is relevant to remember the aforementioned variables that could prevent professionals from suffering compassion fatigue, those being able to control their own workload, social self-care, and coping with death as they are key to professionals’ quality of life and a necessary requisite for them to be able to provide compassionate care for their patients.

Regarding the relation between professional quality of life and well-being, results have shown that compassion satisfaction and burnout were statistically significant predictors of well-being, with their relationships with it being positive and negative, respectively. These results are in line with those found by other authors, who have found that there is a relationship between the professionals’ quality of life and their well-being [14,33,37,40,42,43]. In this sense, the more compassion satisfaction, the lower the burnout and compassion fatigue, which results in greater well-being. It is important to note that, even though in this study, compassion fatigue was not shown as a significant predictor of well-being, the relationship obtained via the model is still relevant and the lack of potency of the study could be behind these results. Given this, more studies with greater sample sizes and predictive capacity that can provide more insight into this matter will be welcomed.

Finally, it is worth mentioning that, whereas a great proportion of variance of well-being and burnout was explained by the model (around 50%), this was not the case for compassion fatigue, compassion satisfaction, and compassion. This means there are some variables that have not been taken into account in current research that could be affecting compassionate care. In this sense, other inner resources, such as self-compassion or mindfulness, have proved to be connected to compassion and compassion satisfaction [14], and therefore, future research studying how these inner resources affect professionals’ compassionate care would be welcomed.

### 4.1. Limitations of the Study

There are some limitations in the current study, for example, the non-aleatory sampling of the subjects, which can affect the representativeness of the sample. In this same sense, the total size of the sample was limited, a factor that could also account for the lack of potency in the analyses and, thus, in the generalizability of the obtained results. One example of this is the relationship between compassion fatigue and well-being that was found in the model, which, although of a respective size, did not reach statistical significance. It is also worth noting that the distribution between genders was not equal, with 77.2% being women (*n* = 186) and 22.8% being men (*n* = 55), which is a really small subgroup. Apart from this, the cross-sectional nature of the study prevents us from establishing and exploring causal relations between the variables included. In this sense, although the directionality of the relationships between variables was taken from the previous literature, the lack of longitudinal data makes it difficult to establish causal relationships. For example, the negative relation found between workload and burnout is assumed to be in the direction that the more workload the professional has, the more burned they will be. However, it could be the other way around, with burned professionals ending up in lower job positions that carry a greater workload. To address this limitation, longitudinal studies would be welcomed. Another limitation of the study is the specific collective chosen, Spanish palliative care workers. It can be that the politics and dynamics that dictate these professionals’ work may not be extrapolatable to other groups of palliative care workers outside Spain and, even inside of Spain, to the more general population of healthcare professionals as a whole. Additionally, response veracity was not assessed. This is a well-known bias when undertaking survey designs so the results of this research should be interpreted taking this fact into account. Finally, the last limitation consists of the lack of information about the professionals’ workplace (i.e., which hospital or healthcare center), as the internal policies and working methods are different between institutions may vary and can affect the way of working that professionals have. These variables could have had an influence on many of the studied variables in this investigation, such as, for example, the professional quality of life.

### 4.2. Future Lines of Research

For future investigations, it would be highly recommended to carry out studies with larger sample sizes and more participants of some specific subgroups, such as men, to be able to better generalize the obtained results. Longitudinal studies would also be welcomed, as they allow researchers to observe changes in the variables via different time sets and to understand the causal relationships that take place between them. Further investigating the relationship between workload and well-being would also be vital as it would enable a greater understanding of how workload affects the compassionate care of healthcare providers. Equally as important, qualitative studies that deepen into some of the most controversial relationships found (for example, gender’s effect or the professionals’ well-being) or in the conceptualizations of terms such as compassionate care and well-being of professionals would also be of great value to improve our understanding of the processes included in this study. Another research proposal could be to study the self-care processes and well-being of professionals at an international level, with larger samples composed of workers from different countries, as this type of study would allow researchers to explore the influence of the different cultural values and dynamics on the studied processes. It would also be enriching if interventions based on an improvement in coping with death were to be designed, as these could help increase the palliative care professionals’ compassionate care and well-being while at the same time providing evidence of how these resources (i.e., coping with death) can affect the latter mentioned processes.

## 5. Conclusions

Compassion and professional quality of life, as it has been seen throughout the scientific literature, are fundamental pieces that enable healthcare workers to provide quality care for their patients and their respective families and, moreover, for the well-being of palliative care professionals. Although gender did not have a significant role in the prediction of the aforementioned compassionate care and well-being of the palliative care professionals as differences were found between men and women, age was shown to be a protective factor for the professional quality of life, with older workers experiencing lower levels of exhaustion/burnout and more compassion satisfaction. The results obtained in this investigation also point out that the professionals’ workload and their control over it (specifically this latter) are variables of great importance when the aim is to either maintain or improve the professionals’ ability to provide compassionate care. More specifically, the greater the control over the own workload, the more compassionate care increases in all of its dimensions, namely increases in compassion and compassion satisfaction while compassion fatigue and burnout decrease.

On the other hand, internal resources of self-care and the ability to cope with death also enlarge the professionals’ ability to provide compassionate care. Compassion was found to be closely related to coping with death. Self-care and coping therefore are essential to palliative care workers and their respective well-being, as they reduce burnout and compassion fatigue. Although no specific relationship between coping with death and well-being was found, it was observed that this variable improved the professional quality of life. This evidence could point out the existence of an indirect relationship between coping with death and well-being mediated by the professional quality of life: coping with death produces an increase in compassion, and, through this, compassion satisfaction increases and compassion fatigue decreases. It is possible that via these effects, coping with death is able to affect well-being.

The results obtained in the study also allow us to conclude that the changes in teamwork that palliative care workers suffered during the COVID-19 pandemic did indeed affect their professional quality of life. In this same sense, having had to provide care for COVID-19 patients was not shown as an important factor in the professionals’ ability to display compassionate care and well-being. A lack of the necessary material resources during the pandemic was shown to contribute to an increase in burnout levels. Finally, it was also demonstrated that the professional quality of life had a predictive role over well-being, specifically the dimensions of compassion satisfaction and burnout.

## 6. Implications for Practice

Having a healthy lifestyle and a social support network are key for palliative care professionals to maintain an adequate professional quality of life. In this same sense, internal resources such as coping with death and self-care should be taken into account when considering how to improve the professionals’ health. It is worth noting that the use of training programs throughout the different healthcare centers is not a mere extravagance but rather a requisite for professionals to be able to provide compassionate care and attention. We suggest giving palliative care professionals opportunities to be trained in the aforementioned skills, as they could be essential to maintaining good self-care and dealing with the high-demanding situations in which healthcare professionals are involved while further improving our compassionate care and well-being.

## Figures and Tables

**Table 1:** Participants’ characteristics.

**Categorical Variables**	**Category**	***n***	**%**
Gender	Women	186	77.2
	Men	55	22.8
	Missing	0	0.0
Material resources	Yes	163	67.6
	No	77	32.0
	Missing	1	0.4
COVID-19 patients	Yes	216	89.6
	No	25	10.4
	Missing	0	0.0
**Ordinal Variables**		**Md**	**IR**
Workload		2.00	0.50
Workload control		2.00	0.50
Changes in workload		5.00	0.50
Changes in teamwork		3.00	1.00
**Quantitative Variables**		**M**	**SD**
Age		45.34	10.92
Physical self-care		3.57	1.05
Psychological self-care		2.97	1.14
Social self-care		4.07	0.76
Coping with death competence		3.96	0.63

Notes: Md = median; IR = interquartile range; M = mean; SD = standard deviation.

**Table 2:** Descriptive statistics for the studied variables.

Variable	M	SD	Min	Max	Sk	Kur
Compassion	4.41	0.43	3.20	5.00	−0.55	−0.12
Compassion satisfaction	13.87	1.45	7.00	15.00	−1.16	3.29
Burnout	8.23	2.37	3.00	15.00	0.13	−0.01
Compassion fatigue	6.97	2.09	3.00	15.00	0.62	0.89
Well-being	3.95	0.56	2.25	5.00	−0.54	0.45

Notes: M = mean; SD = standard deviation; Min = minimum score; Max = maximum score; Sk = skewness; Kur = kurtosis.

**Table 3:** *T* test results for gender differences and descriptive statistics for the groups.

Variable	*t*	df	*p*	Cohen’s d	95% CI		Women		Men	
					Inf	Sup	M	SD	M	SD
Compassion	0.79	232	0.44	0.12	−0.18	0.43	4.42	0.45	4.37	0.37
Compassion satisfaction	0.85	237	0.40	0.13	−0.17	0.44	13.91	1.47	13.72	1.39
Burnout	1.33	239	0.18	0.21	−0.08	0.51	8.34	2.31	7.85	2.53
Compassion fatigue	1.37	238	0.17	0.21	−0.09	0.51	7.07	2.08	6.63	2.11
Well-being	1.47	213	0.14	0.24	−0.08	0.55	3.98	0.53	3.85	0.65

Notes: *t* = statistic value; df = degrees of freedom; CI = confidence interval; M = mean; SD = standard deviation.

**Table 4:** Relations between age, workload, workload control, self-care, coping with death competence, changes in workload and changes in teamwork, compassion, professional quality of life, and well-being.

Variable	Compassion	Compassion Satisfaction	Burnout	Compassion Fatigue	Well-Being
Age	−0.02	0.11	−0.19 **	−0.07	0.04
Workload	0.00	−0.02	0.52 **	0.20 **	0.04
Workload control	0.14 *	0.24 **	−0.38 **	−0.26 **	0.12
Physical self-care	0.06	0.16 *	−0.29 **	−0.31 **	0.37 **
Psychological self-care	0.13	0.19 **	−0.14 *	−0.25 **	0.46 **
Social self-care	0.13 *	0.26 **	−0.36 **	−0.38 **	0.55 **
Coping with death competence	0.20 **	0.20 **	−0.16 *	−0.27 **	0.30 **
Changes in workload	0.01	0.04	0.25 **	0.08	0.07
Changes in teamwork	−0.05	0.15 *	−0.17 **	−0.07	0.06

Notes: For quantitative variables, Pearson correlations were calculated; for ordinal variables, Spearman correlations were calculated; * *p* < 0.05; ** *p* < 0.01.

**Table 5:** *T* test results for resource differences and descriptive statistics for the groups.

Variable	*t*	df	*p*	Cohen’s d	95% CI		Yes		No	
					Inf	Sup	M	SD	M	SD
Compassion	0.83	231	0.40	0.12	−0.16	0.39	4.39	0.42	4.44	0.46
Compassion satisfaction	1.32	236	0.19	−0.18	−0.46	0.09	13.95	1.36	13.68	1.63
Burnout	3.42	238	<0.01	0.47	0.2	0.75	7.87	2.42	8.96	2.09
Compassion fatigue	1.64	237	0.10	0.23	−0.04	0.50	6.81	2.22	7.29	1.75
Well-being	0.14	212	0.89	0.02	−0.27	0.31	3.94	0.56	3.95	0.57

Notes: *t* = statistic value; df = degrees of freedom; CI = confidence interval; M = mean; SD = standard deviation.

**Table 6:** *T* test results for attending COVID-19 patients and descriptive statistics for the groups.

Variable	*t*	df	*p*	Cohen’s d	95% CI		Yes		No	
					Inf	Sup	M	SD	M	SD
Compassion	0.97	232	0.33	0.20	−0.21	0.62	4.42	0.43	4.33	0.43
Compassion satisfaction	1.46	237	0.15	0.31	−0.11	0.74	13.91	1.41	13.46	1.72
Burnout	1.68	239	0.10	0.35	−0.06	0.77	8.31	2.38	7.48	2.12
Compassion fatigue	0.65	238	0.52	0.14	−0.28	0.56	7.00	2.04	6.71	2.49
Well-being	1.74	213	0.08	0.38	−0.05	0.80	3.97	0.55	3.76	0.59

Notes: *t* = statistic value; df = degrees of freedom; CI = confidence interval; M = mean; SD = standard deviation.

**Table 7:** Factor loadings for the measurement part of the model.

Compassion	λ	Well-Being	λ
Item 1	0.44	Item 1	0.87
Item 2	0.73	Item 2	0.69
Item 3	0.85	Item 3	0.81
Item 4	0.80	Item 4	0.70
Item 5	0.70	Item 5	0.77
		Item 6	0.80
		Item 7	0.76
		Item 8	0.61

Notes: All the factor loadings were statistically significant (*p* < 0.001).

**Table 8:** Effects of the control variables included in the structural equation model.

Variable	Compassion	Compassion Satisfaction	Burnout	Compassion Fatigue	Well-Being
Age	−0.02	0.13 *	−0.19 ***	−0.07	−0.01
Gender	−0.06	−0.06	−0.02	−0.08	−0.07
Workload	−0.03	0.06	0.35 ***	0.05	0.16 *
Workload control	0.14 *	0.16 *	−0.19 ***	−0.17 **	−0.02
Material resources	−0.08	0.09	−0.16 **	−0.08	−0.04
Changes in workload	−0.03	0.09	0.13	−0.01	0.12
Changes in teamwork	0.01	0.13 *	−0.14 *	−0.06	0.03
Treating COVID-19 patients	0.07	0.05	0.05	0.02	0.06
Physical self-care	−0.06	0.07	−0.17 **	−0.06	0.04
Psychological self-care	0.06	0.04	0.05	−0.10	0.29 ***
Social self-care	0.12	0.04	−0.18 **	−0.35 ***	0.43 ***
Coping with death competence	0.22 **	0.06	−0.07	−0.17 **	0.10

Notes: For quantitative variables, Pearson correlations were calculated; for ordinal variables, Spearman correlations were calculated; * *p* < 0.05; ** *p* < 0.01; *** *p* < 0.001.

## Data Availability

The data that support the findings of this study are available from the corresponding author upon reasonable request.
